# Commentary: Nurse staffing, self-efficacy, and the challenge of preventing patient falls

**DOI:** 10.1177/17449871251392291

**Published:** 2025-11-17

**Authors:** Sarah Bekaert

**Affiliations:** Associate Professor, Faculty of Humanities and Social Sciences, Oxford Brookes University, Oxford UK

**Commentary to:** Nurses’ knowledge, self-efficacy and workplace support in fall prevention: a cross-sectional study (Batiha, 2025).

Patient falls remain a persistent and costly challenge in healthcare systems worldwide. Despite decades of preventive initiatives, hospitals continue to report consistent fall rates, with the World Health Organization ranking falls as the second leading cause of unintentional injury deaths globally (WHO, 2021). This commentary explores the study by Batiha et al. (2025) carried out in Jordan, where high patient-to-nurse ratios, underreporting, and limited professional development opportunities undermine consistent fall prevention practices. Nurses, as the frontline providers of inpatient care, are central to fall prevention efforts. However, nurses ability to carry out nursing care depends not only on knowledge and self-efficacy but also on supportive workplace structures that empower them to translate skills into practice. This is a perennial concern in nursing across all specialisms and globally, as seen for example in this journal in studies by Vaismoradi et al. (2012) who explored Iranian nurses’ experiences and perspectives on how to provide safe care in clinical practice, and Beardsmore and McSherry (2017) who looked at organisational culture and the impact on the delivery of compassionate quality care. I have also discussed previously in this journal how cognitive load affects nurses’ ability to adhere to clinical guidelines, a relevant consideration in fall prevention practices (Bekaert, 2025).

## Study summary

Batiha et al. (2025) adopted a quantitative, cross-sectional design to investigate the relationship between fall prevention knowledge, self-efficacy and structural empowerment among 78 registered nurses in four Jordanian hospitals. Data were gathered using three validated instruments: the Fall Prevention Knowledge Test (FPKT) (Dykes et al., 2023), the Self-Efficacy for Preventing Falls Nurse Scale (SEPFN) (Dykes et al., 2011) and the Conditions of Work Effectiveness Questionnaire II (CWEQ-II) (Laschinger et al., 2000). The findings revealed a significant positive association between nurses’ knowledge and self-efficacy (*r* = 0.294, *p* = 0.017), particularly among those with advanced education and over 10 years of clinical experience. Surprisingly, structural empowerment did not strengthen this relationship. Instead, a negative correlation emerged between self-efficacy and perceived institutional support (*r* = −0.249, *p* = 0.047), suggesting a disconnect between organisational support structures and the needs of confident and/or experienced nurses. Although units with higher nurse self-efficacy reported reduced fall rates, the difference was not statistically significant.

## Critical insights

The study offers several important contributions. Firstly, it reinforces evidence that knowledge and confidence are mutually reinforcing: nurses with stronger educational backgrounds and longer experience are more effective in fall prevention. Secondly, it raises an intriguing paradox. Although theory suggests structural empowerment enhances performance (Kanter, 1993), these findings indicate that experienced nurses may perceive institutional support as misaligned with their professional autonomy. The authors reflect that this disconnect may reflect the rigid, resource-limited environments of Jordanian hospitals, where support mechanisms are often underdeveloped or poorly tailored to frontline realities. However, they note that equally important is the role of workload and burnout – a recurring and escalating area of exploration in nursing research (as explored in this journal e.g. by Al Yahyaei et al., 2022; Kiratli and Duran, 2024). The study identifies these as critical barriers that undermine self-efficacy and perceptions of support. This echoes wider international evidence linking high patient-to-nurse ratios and chronic staff shortages with poorer safety outcomes (Ball and Catton, 2011; Dall’Ora et al., 2022; Yanchus et al., 2017). In lower-resource settings such as Jordan, addressing these systemic constraints is as vital as enhancing individual knowledge and confidence.

## Implications for practice and policy

For nursing practice, the study highlights the need to strengthen knowledge and self-efficacy through simulation-based training, mentorship, and continuing professional development. Simulation and case-based learning provide opportunities to reinforce both cognitive and practical competencies in fall prevention. For policymakers and hospital leaders, the findings underscore the urgency of addressing workload pressures through staffing adjustments, flexible scheduling and task redistribution. Institutional support should be flexible and responsive, providing experienced nurses with opportunities for leadership, decision-making, and peer mentoring rather than rigidly standardised programmes.

## Future directions

Although this study was strong in that it used validated tools and diverse inpatient units, its small sample size, reliance on self-reporting, and focus on four hospitals limit generalisability – which the authors acknowledge. Future recommendations are for research that adopts multi-site, longitudinal designs to better capture causal relationships and explore how institutional factors interact with knowledge and self-efficacy over time. Also mixed-methods approaches which could also deepen understanding of why nurses with high self-efficacy feel less supported, revealing nuances that quantitative data alone cannot capture.
